# Vernakalant for Cardioversion of Recent-Onset Atrial Fibrillation in the Emergency Department: The SPECTRUM Study

**DOI:** 10.1159/000526831

**Published:** 2022-11-10

**Authors:** Johan-Emil Bager, Alfonso Martín, José Carbajosa Dalmau, Alexander Simon, José L. Merino, Beate Ritz, Juha E.K. Hartikainen

**Affiliations:** ^a^Department of Molecular and Clinical Medicine, Institute of Medicine, Sahlgrenska Academy, University of Gothenburg, Gothenburg, Sweden; ^b^Department of Emergency Medicine, Sahlgrenska University Hospital, Gothenburg, Sweden; ^c^Emergency Department, University Hospital of Móstoles, Madrid, Spain; ^d^Emergency Department, General University Hospital of Alicante, Alicante, Spain; ^e^Department of Emergency Medicine, Medical University of Vienna, Vienna, Austria; ^f^Department of Cardiology, La Paz University Hospital, IDIPAZ, Universidad Autónoma de Madrid, Madrid, Spain; ^g^Medical Information Department, ADVANZ PHARMA Switzerland Sàrl, Geneva, Switzerland; ^h^Heart Center Department, Kuopio University Hospital, Kuopio, Finland; ^i^University of Eastern Finland, Kuopio, Finland

**Keywords:** Atrial fibrillation, Emergency department, Antiarrhythmic drugs, Safety, Vernakalant

## Abstract

**Introduction:**

Intravenous vernakalant is a therapeutic option for symptomatic, recent-onset atrial fibrillation (AF). This secondary analysis from the large SPECTRUM study assessed the safety and effectiveness of vernakalant when used in the emergency department setting (ED group) or in an inpatient hospital setting (non-ED group).

**Methods:**

This post hoc analysis of the international, observational, post-authorization SPECTRUM study included 1,289 and 720 recent-onset AF episodes in adults in the ED and non-ED groups, respectively. Safety endpoints included the evaluation of pre-defined health outcomes of interest (HOIs) and other serious adverse events (SAEs) during vernakalant treatment and during the first 24 h after the last infusion. Effectiveness endpoints comprised the rate of successful vernakalant cardioversion, the time from the start of the vernakalant infusion to cardioversion, and the length of hospital stay. Data were analysed using descriptive statistics.

**Results:**

The safety profile of vernakalant was similar in the ED and non-ED groups. In the ED group, 12 pre-defined HOIs were reported in 11 patients (0.9%); all but one occurred within 2 h after start of the first infusion. These events comprised nine significant bradycardia cases, of which one was associated with transient hypotension and three with sinus arrest, and 2 cases of atrial flutter with 1:1 conduction. Five other SAEs were reported. All patients with vernakalant-related events recovered without sequelae. No Torsade de Pointes, ventricular fibrillation, or deaths occurred. Successful cardioversion was reported in 67.8% (95% confidence interval: 65.2–70.4) and 66.4% (62.5–70.1) of episodes, with a median time to conversion of 11.0 and 10.0 min in the ED and non-ED groups, respectively. Patients had a median length of hospital stay of 7.4 h and 17.1 h in the ED and non-ED groups, respectively.

**Conclusion:**

Intravenous vernakalant was well tolerated with similar cardioversion rates in patients treated in the ED or non-ED setting and does not require admission to a coronary care unit or intensive care unit. First-line treatment with vernakalant could allow an early discharge in patients with recent-onset AF treated in the ED.

## Introduction

Symptomatic, recent-onset atrial fibrillation (AF) is a common condition in the emergency department (ED) [1]. However, the treatment algorithm varies largely between the USA and Europe, with lower hospital admission rates in Europe due to the higher use of immediate cardioversion in an ambulant setting [2, 3, 4]. In haemodynamically stable patients, both electrical and pharmacological cardioversion are safe and effective options for rhythm control management [5, 6]. While electrical cardioversion is highly effective in restoring sinus rhythm (SR) [5, 7], pharmacological cardioversion may be more practical for the treatment of patients in the ED since it does not require fasting and sedoanalgesia [2, 5, 6]. Therefore, pharmacological cardioversion is often chosen as first-line treatment for patients with recent-onset AF, mainly those with no or mild-to-moderate structural heart disease (i.e., excluding patients with recent acute coronary syndrome, heart failure [HF], reduced ejection fraction, severe left ventricular hypertrophy, or advanced valve disease) [5, 6, 7]. Recommended options in Europe include intravenous vernakalant (excluding patients with recent acute coronary syndrome, severe HF, or severe aortic valve stenosis), flecainide, or propafenone (excluding patients with severe structural heart disease). Intravenous amiodarone is another recommended option in patients with HF or structural heart disease if a delayed cardioversion is consistent with their clinical situation [6]. Despite being frequently used in the ED, amiodarone has a slower onset of action and is less effective at achieving cardioversion compared with other antiarrhythmic drugs, although it does offer rate control [2, 5, 8]. Previous studies have shown that intravenous vernakalant, a fast-acting and effective antiarrhythmic drug for pharmacological cardioversion, has a favourable safety profile and induces rapid cardioversion in a high proportion of patients with recent-onset AF [9, 10, 11, 12]. Vernakalant preferentially targets atrial potassium channels and rate- and depolarization-dependent sodium channels [13]. Despite its approval in 2010 by the European Union for cardioversion of recent-onset AF (less than 7 days in duration, or for post-operative AF less than 3 days in duration), vernakalant is currently not widely used and some uncertainty about its safety remains [13]. While the rapid cardioversion obtained with vernakalant is a major asset for patients with recent-onset AF treated in the ED to potentially avoid hospital admission, the high staff turnover rates and operational constraints in this setting could present challenges for its appropriate use in this population. We conducted a post hoc analysis of the SPECTRUM (Surveillance of Pharmacologic thErapy for Cardioversion in aTrial fibrillation Registry Using intravenous treatMent) study in patients with symptomatic, recent-onset AF who were treated in the ED to evaluate the safety and effectiveness of vernakalant for cardioversion in this setting.

## Materials and Methods

### Study Design, Setting, and Population

SPECTRUM was an international, multicentre, observational, post-authorization study, which was conducted in 53 hospitals in Austria, Denmark, Finland, Germany, Spain, and Sweden to evaluate the safety, the adherence to the approved indications and dosages, and the effectiveness of vernakalant in symptomatic patients with recent-onset AF in routine clinical practice. The results of the main analysis of the SPECTRUM study, comprising 2009 treatment episodes, have been published elsewhere [14]. Here, we report the results of a post hoc analysis of the patients treated in the ED. Eligible patients were adults with recent-onset AF occurring between February 29, 2012, and April 3, 2018, who received one or two vernakalant infusions in the ED (ED group) or in an inpatient hospital setting (non-ED group), gave informed consent for study participation, and did not participate in an investigational clinical trial within 30 days prior to enrolment.

**Fig. 1:**
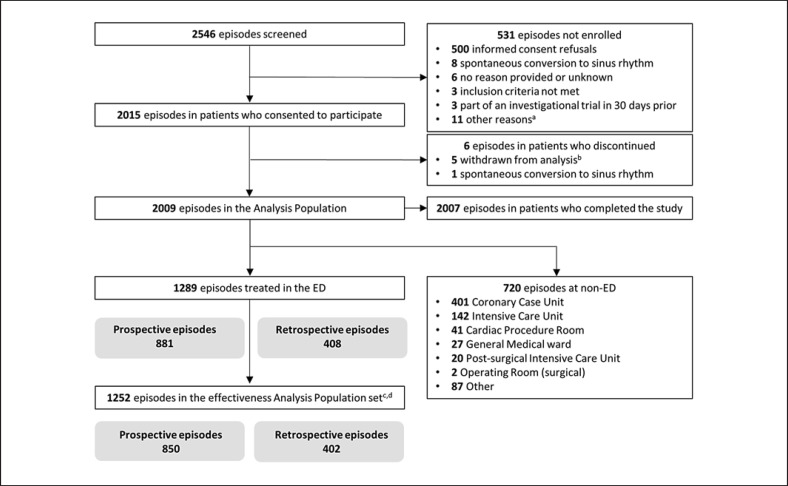
Enrolment diagram for patients included in the study, with a focus on patients receiving intravenous vernakalant in the ED. ED, emergency department; Prospective episodes, episodes in patients who were prospectively enrolled in the study; Retrospective episodes, episodes in patients who were retrospectively enrolled in the study. ^a^Other reasons include, but are not limited to, the following: ejection fraction 30–35%, preference for direct current cardioversion, missing information regarding start of atrial fibrillation. ^b^The patients were withdrawn for the following reasons: source data not verified by study monitor (2 patients without confirmed vernakalant administration and 2 patients from an unresponsive study site), no proof of signed informed consent form (1 patient). ^c^Effectiveness analysis population set includes all treatment episodes among the enrolled eligible patients who signed the informed consent form and received vernakalant intravenously except those who received another cardioversion therapy within 90 min of start of the first vernakalant administration (i.e., electrical or pharmacologic cardioversion). ^d^Patients with single or multiple enrolments.

Data were collected prospectively for most patients, except for a minority who received vernakalant after April 13, 2013, and were included retrospectively following approval of a protocol amendment by the European Medicines Agency due to slow enrolment (Fig. 1). The physicians decided to treat patients with vernakalant independently of the study.

**Fig. 2:**
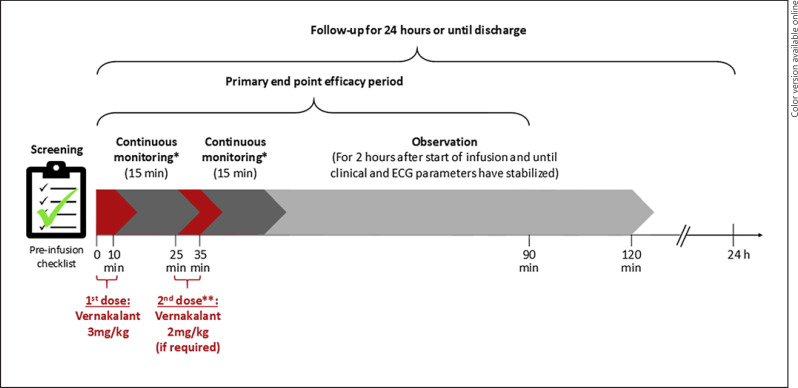
Study design. ECG, electrocardiogram. *Patients should be carefully monitored for any signs or symptoms of a sudden drop in blood pressure or heart rate, with or without symptomatic hypotension or bradycardia, during and for at least 15 min after completion of the infusion. **If conversion to sinus rhythm did not occur during or within 15 min after the first infusion, a 2 mg/kg second infusion should be administered over 10 min.

As per European label, vernakalant is administered with up to two infusions of 10 min, with a monitoring period of 15 min after each infusion, and an observation period of at least 2 h (Fig. 2). The recommended doses for the first and second infusions are 3.0 mg/kg and 2.0 mg/kg, respectively. The study follow-up period was 24 h after the last vernakalant infusion or until the end of the medical encounter, whichever came first.

This study was mandated and approved by the European Committee for Medicinal Products for Human Use. The protocol was approved by the appropriate local research Ethics Committees for all participating centres and the study was conducted in accordance with applicable national and local regulations, accepted standards for Good Clinical Practice, Guidelines for Good Pharmacoepidemiology Practices, and the Declaration of Helsinki. Informed written consent was obtained from all patients. The study is registered at http://www.clinicaltrials.gov (NCT01370629) and http://www.encepp.eu (EUPAS2078). The conduct and reporting of this study were supported by ADVANZ PHARMA Switzerland Sàrl.

### Study Protocol

In this post hoc analysis of the SPECTRUM study, the safety endpoints included the incidence of clinically significant pre-defined health outcomes of interest (HOIs) and other severe adverse events (SAEs) in patients with recent-onset AF who received vernakalant in the ED and non-ED groups. The HOIs included significant hypotension (systolic blood pressure <90 mm Hg, requiring treatment with vasopressors), ventricular arrhythmias (sustained ventricular tachycardia [VT] with a ventricular heart rate >120 beats per minute with a duration >30 s, VT that required intervention with either electrical cardioversion or antiarrhythmic drugs, Torsade de Pointes with a duration >10 s, or ventricular fibrillation of any duration), atrial flutter (AFL with 1:1 atrioventricular conduction with a duration >10 s and a ventricular rate >200 beats per minute), or bradycardia (requiring electrical pacing or any other SAE reports involving bradycardia, including sinus arrest and asystole).

Successful cardioversion and time to conversion were determined for all episodes according to the following definition: conversion to SR for at least 1 min within 90 min from the start of the first vernakalant infusion. Time to conversion was also determined for vernakalant conversion failures, when other treatment such as electrical cardioversion, intravenous class I/III antiarrhythmics, oral propafenone, or flecainide were used. Proportions of patients treated in accordance with the approved indications and contraindications for vernakalant infusions [6, 15], the use of the pre-infusion checklist (online suppl. S1; for all online suppl. material, see www.karger.com/doi/10.1159/000526831), as well as the duration of hospital stay and AF episodes were also evaluated.

### Key Outcome Measures

Data were collected from medical records (all patients) and from supplemental data collection forms (prospectively enrolled patients [68.3% and 97.1% of patients in the ED and non-ED groups, respectively]). Patient demographics, presenting conditions, clinical parameters, medical history, concomitant medication, AF episode, laboratory measures, and other antiarrhythmic therapies during hospitalization were recorded. The AF episode duration was calculated as the time between the patient-reported time of symptom onset and the start of the first vernakalant infusion.

### Data Analysis

This was a descriptive analysis. Patients could be enrolled more than once if they presented on multiple occasions with unique AF episodes. Each episode was treated independently and could correspond to one or two vernakalant infusions. Effectiveness analyses were performed on all episodes and on a pre-defined effectiveness population (online suppl. S2). Denominators in the results section reflect treatment episodes with available data and can therefore vary depending on the analysis.

Categorical variables were summarized by numbers and percentages of patients. The 95% confidence intervals (CIs) were calculated using exact methods described by Clopper and Pearson. Continuous variables were summarized using descriptive statistics (mean, standard deviation, median, interquartile range [IQR]). The cumulative incidence of HOIs was calculated as the number of episodes in patients with a HOI starting during the follow-up period, divided by the total number of episodes, multiplied by 100. All analyses were performed using Statistical Analysis System (SAS) version 9.2.

### Patient and Public Involvement Statement

Patients or the public were not involved in the design, conduct, reporting, or dissemination plans of our research.

## Results

### Study Population and Treatment

A total of 1,289 vernakalant treatment episodes were administered in 1,120 unique patients with recent-onset AF in the ED group (Fig. 1). Among the 1,120 unique patients, 995 patients had a single inclusion and 125 had multiple inclusions in the analyses for distinct AF episodes (online suppl. S3). No patient discontinued the study. Data were collected prospectively for 881 (68.3%) treatment episodes and retrospectively for 408 (31.7%) treatment episodes with vernakalant after April 13, 2013. In the non-ED group, a total of 720 vernakalant treatment episodes were administered in 664 unique patients. Most of the episodes were in patients admitted to coronary care unit (401 episodes [55.7%]) and intensive care unit (142 episodes [19.7%]), and there were 97 post-cardiac surgery episodes.

Baseline patient characteristics of all treatment episodes are summarized in Table 1. In the ED group, the median (IQR) duration of the AF episode prior to treatment was 9.1 h (4.8, 19.3) and in 92.7% of episodes, patients were treated within 48 h from the onset of symptoms. Baseline characteristics of patients in the ED and non-ED groups were generally similar, except for the longer duration of the current AF episode in the non-ED group (median [IQR]: 15.8 h [7.7, 35.1]).

### Safety Results

In the ED group, 12 pre-defined HOIs were reported for 11 episodes (0.9% [95% CI: 0.4–1.5]) (Table 2). Eleven of these HOIs occurred within 2 h after vernakalant infusion start. All patients with HOIs recovered without sequelae.

**Fig. 3:**
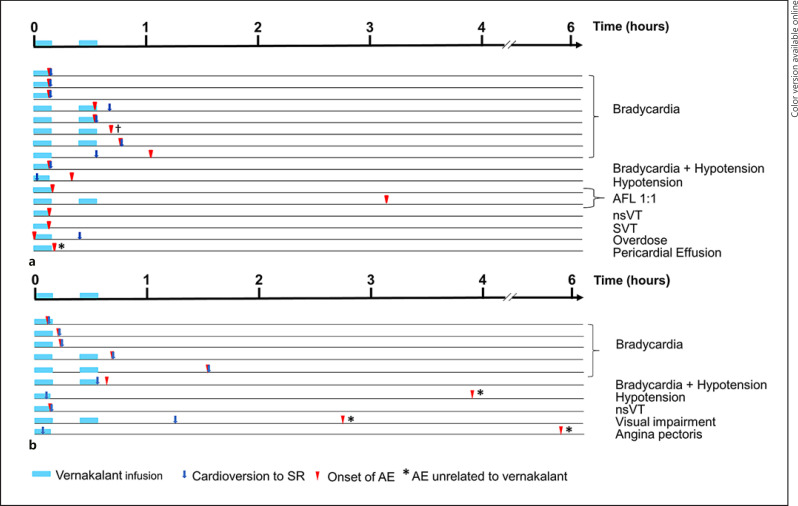
Time of vernakalant infusion, cardioversion, onset of HOIs, and SAEs in AF episodes in the ED group (**a**) and the non-ED group (**b**). AF, atrial fibrillation; ED, emergency department; AFL, atrial flutter; nsVT, non-sustained ventricular tachycardia; SVT, supraventricular tachycardia; SR, sinus rhythm; AE, adverse event. ^†^This patient experienced bradycardia 42 min after the vernakalant infusion without cardioversion and underwent successful electrical cardioversion 7 h later.

The most common HOI was significant bradycardia (9 cases [0.7%]). All bradycardia episodes occurred within 2 h after vernakalant infusion start, 6 of them occurred concurrently with cardioversion, and none required vasopressors or electrical pacing (Fig. 3a). Three of the bradycardia episodes were associated with a sinus arrest that resolved spontaneously within a few seconds. Two cases (0.2%) of AFL 1:1 were reported and both patients underwent electrical cardioversion. Additional details on the AFL 1:1 events are presented in online suppl. S4. No Torsade de Pointes, ventricular fibrillation, or deaths occurred.

Five other SAEs, including 1 case of hypotension and 1 case of non-sustained VT, were reported (Table 2). All patients recovered without sequelae, except 1 patient with a pericardial effusion SAE (presumably not related to vernakalant) who developed tachycardia-induced HF. The most frequently reported non-serious AEs were sneezing, non-1:1-conducted AFL, and dysgeusia.

The safety profile of vernakalant was similar in the ED and non-ED groups. In the non-ED group, 6 significant bradycardia events [0.8%] were reported, of which 1 was in a patient with a simultaneous event of significant hypotension [0.1%] (Fig. 3b). No HOIs or other SAEs were reported in patients who received antiarrhythmic drugs or electrical cardioversion after vernakalant infusion.

### Effectiveness Results

In episodes with available information, patients received one vernakalant infusion in 777/1,270 (61.2%) and 424/720 (58.9%) episodes and two infusions in 493/1,270 (38.8%) and 296/720 (41.1%) episodes in the ED and non-ED groups, respectively. In the ED group, infusions were discontinued prematurely in 36 (2.8%) episodes, the most common reason (21 episodes) being conversion to SR (other reasons were 5 mild AEs [sneezing, nausea, metal taste in the mouth], 3 non-sustained VT, 1 case of sinus bradycardia, 1 case of AFL, 2 hypotension, and 3 unspecified/unknown).

Cardioversion data were missing for 8 and 98 episodes, and successful cardioversion was reported in 869/1,281 (67.8% [95% CI: 65.2–70.4]) and 413/622 (66.4% [62.5–70.1]) episodes with available cardioversion data in the ED and non-ED groups, respectively. The results obtained in the effectiveness population are presented in the online suppl. S2.

**Fig. 4:**
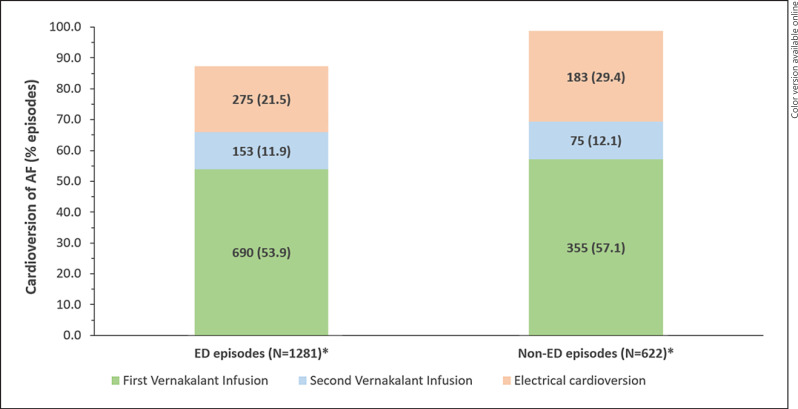
Proportion of episodes in patients who were successfully cardioverted after the first or the second dose of vernakalant or with a subsequent electrical cardioversion in the ED and non-ED groups. ED, emergency department; AF, atrial fibrillation; N, number of patients with available cardioversion results. *Cardioversion data were missing for 8 and 98 patients in the ED and non-ED groups, respectively.

The number of infusions and the actual time to conversion were available for 843/869 and 410/413 episodes for which successful cardioversion was reported in the ED and non-ED groups, respectively. Among these episodes, 690/843 (81.9%) and 335/410 (81.7%) episodes were in patients who had received one vernakalant infusion and 153/843 (18.1%) and 75/410 (18.3%) episodes in patients who had received two infusions in the ED and non-ED groups, respectively (Fig. 4). The median (IQR) time to conversion was 11.0 (8.0, 20.0) and 10.0 (7.0, 23.0) min in the ED and non-ED groups, respectively.

### Adherence

In the ED group, the pre-infusion checklist (online suppl. S1) was used in 688/762 (90.3%) episodes with available information on its use. Vernakalant was administered in 36/1,289 (2.8%) and 65/720 (9.0%) episodes in patients with at least one contraindication in the ED and non-ED groups, respectively (for further information, see online suppl. S5).

### Length of Hospital Stay

The median (IQR) length of hospital stay for patients with a recent-onset AF episode who received vernakalant in the ED was 7.4 h (5.1, 13.4) and only 12.1% of patients were in hospital for ≥24 h. In the non-ED group, the median (IQR) length of hospital stay was 17.1 h (7.5, 52.2) and 42.9% of patients were in hospital for ≥24 h.

### Use of Other Antiarrhythmic Agents and Electrical Cardioversion to Restore SR

In the ED group, other class I or class III antiarrhythmic agents were administered intravenously for pharmacologic cardioversion within 90 min after the start of first vernakalant infusion in 4/1,268 (0.3%; only amiodarone) episodes with available information. Oral class I and class III antiarrhythmic agents were administered for pharmacologic cardioversion subsequent to vernakalant infusion in 2/1,268 (0.2%; flecainide in both cases) and 5/1,268 (0.4%) episodes with available information.

At least one electrical cardioversion was performed after the start of vernakalant infusion to restore SR in 293/1,268 (23.1%) and 189/715 (26.4%) episodes with available information in the ED and non-ED groups, respectively. In 33 episodes in the ED group and 35 episodes in the non-ED group, electrical cardioversion was performed within 90 min after the vernakalant infusion start. A successful restoration of the SR was achieved with electrical cardioversion in 275/297 (92.6%) and 183/193 (94.8%) episodes in the ED and non-ED groups, respectively (Fig. 4).

## Discussion

Management of AF in the ED varies greatly and is subject to specific challenges to prevent unnecessary hospital admissions while ensuring safe transition of care after acute treatment. This post hoc analysis of the SPECTRUM post-authorization study, which is the hitherto largest study conducted on the use of vernakalant in patients with recent-onset AF, demonstrated that the safety profile and effectiveness of vernakalant were similar in patients treated in the ED and in an inpatient hospital setting.

While the demographic characteristics of patients undergoing pharmacological cardioversion in the ED were generally similar in the SPECTRUM and the previously published RHYTHM-AF study [2], the prevalence of HF history was lower in the SPECTRUM study (2.6 vs. 10.5%), reflecting the adequate patient selection for vernakalant. The high proportion of episodes treated with vernakalant in the ED within 48 h after the onset of symptoms (92.7%) is consistent with the general use of vernakalant and other antiarrhythmic agents [10, 16, 17]. Indeed, a short duration (<12 h) of the AF episode is a well-known predictor for successful pharmacological cardioversion [10, 16]. Since the median duration of AF episodes prior to treatment was 9.1 h and the median time to conversion 11.0 min in vernakalant responders treated in the ED, a large proportion of patients could be cardioverted within 12 h of AF onset, a time during which the incidence of thromboembolic events was shown to be specifically low [6, 18].

In our analysis, pre-defined HOIs were reported in approximately 1.0% of episodes of recent-onset AF in patients treated in the ED. The low incidence of significant bradycardia and hypotension was reassuring; the single significant hypotension (transient and treated without need of vasopressors) was associated with bradycardia and most (7/9 [77.8%]) bradycardia events were associated with rhythm changes (a phenomenon which is frequently observed with cardioversion regardless of the method used) [17]. Three bradycardia cases were associated with sinus arrest and 2 cases of significant AFL were also reported, but there were no cases of Torsade de Pointes or ventricular fibrillation and all pre-specified HOIs resolved without sequelae. The incidence of other SAEs was also low (0.4%), and all patients with vernakalant-related events recovered without sequelae. These results confirm that vernakalant is well tolerated when administered to appropriately selected patients presenting in the ED with recent-onset AF [15, 19].

In patients who received vernakalant in the ED, all pre-defined HOIs, except one episode of significant AFL, were reported within 2 h after the infusion start, confirming that most patients who converted successfully could be discharged from the hospital after 2 h of monitoring if clinical and electrocardiogram parameters were stable [20]. This is an important advantage of vernakalant compared with other antiarrhythmic drugs, which could allow a reduction of hospital stay durations and related costs [20]. Most patients who received vernakalant in the ED were discharged early from the hospital (<24 h), and the median length of their hospital stay was 7.4 h, consistent with previously published results on the use of vernakalant in patients with recent-onset AF treated in the ED (online suppl. S6) [10, 16, 21].

In our analysis, a successful cardioversion was observed within 90 min after the start of the vernakalant infusion in 67.8% of episodes treated in the ED, consistent with the results of the overall SPECTRUM study population [14]. These results are similar to other studies where patients with recent-onset AF received vernakalant in the ED (66–84% effectiveness on restoring SR; online suppl. S6) [10, 16, 20, 21, 22, 23]. However, lower SR restoration rates (approximately 50%) were observed in patients with recent-onset AF in previous phase 3 clinical trials, in which AF durations up to 48 h [24] or 7 days [25, 26] were accepted. Besides the reduced risk of thromboembolic complications, the rapid onset of vernakalant action could also be important to prevent atrial remodelling and progression to permanent AF, to improve patients' quality of life, and to facilitate their early discharge from the hospital [27]. Even though a previous study has shown that a wait-and-see strategy was non-inferior to early cardioversion in achieving SR at 4 weeks among selected patients with recent-onset AF [28], the early symptom relief offered by vernakalant may be particularly important for the often highly symptomatic patients treated in the ED [1, 2, 29].

This study reports that electrical cardioversion was used in 293 (23.1%) episodes in patients treated in the ED who did not convert after one or two vernakalant infusions. In most of them (92.6%), restoration to SR was successfully attained after electrical cardioversion. These results suggest that while vernakalant is a well-tolerated and effective first-line treatment for patients presenting in the ED with recent-onset AF and without acute hemodynamic instability [6, 30], electrical cardioversion can be used as second-line option for those who fail to restore SR after two vernakalant infusions [3, 7]. Of note, electrical cardioversion requires prior fasting, which may delay such treatment for many patients presenting to the ED.

According to the present analysis, the pre-infusion checklist was used in most episodes (90.3%) and vernakalant was less frequently administered to patients with contraindications (2.8 vs. 9.0%) in the ED compared to inpatient hospital settings. This suggests that the use of the pre-infusion checklist helps to ensure appropriate patient selection and treatment, particularly in the acute setting.

While other agents are recommended for pharmacological cardioversion by the European Society of Cardiology, their use is often limited by the delayed onset of action and side effects [6, 31]. A randomized phase 3 study has shown that in patients with recent-onset AF, vernakalant was superior to the widely used amiodarone in terms of cardioversion rate (51.7 vs. 5.2% within 90 min) [24]. Vernakalant was also more effective in achieving rapid cardioversion and was associated with shorter hospital stays than flecainide in a non-randomized study [9]. A randomized study has also shown that vernakalant was superior to ibutilide in terms of 90-min cardioversion rate (69 vs. 43%) and median time to conversion (10 min vs. 26 min) [22]. Another randomized study did not confirm the higher cardioversion rate in patients treated with vernakalant versus ibutilide (52.8 vs. 52.4%), but shorter mean times to conversion (11.8 min vs. 33.9 min) and shorter mean hospital stays (17.6 h vs. 41.1 h) were reported in patients who received vernakalant [32]. In another single-centre study, no significant difference in cardioversion rates was observed in patients with recent-onset AF who received ibutilide or vernakalant in the ED (68.2 vs. 79.4%) [12]. Of note, unlike vernakalant, ibutilide has been associated with Torsades de Pointes [33].

The limitations of this analysis should be acknowledged. Its post hoc nature represents a limitation as results were descriptive and should be cautiously interpreted. Additionally, the retrospective enrolment of almost one third of patients treated in the ED, the dependence on data availability in their records, and the fact that patients could be enrolled more than once, with each episode treated independently, could also influence the results. Nevertheless, the impact of these factors was probably limited, without significantly changing the external validity of present results. Indeed, most episodes (77.2% [995/1,289]) were reported in patients who were only included once, and baseline characteristics of prospectively and retrospectively enrolled patients were similar, except a numerically slightly shorter duration of the AF episode in retrospective patients. Another limitation was the absence of a placebo control group, which does not allow to differentiate spontaneous conversion from vernakalant-induced cardioversion. Nevertheless, from a clinical standpoint, our results support the use of vernakalant to treat patients with recent-onset AF since it was well tolerated, effective, and time saving, which is of utmost importance when planning management strategies in clinical practice. The 24-h follow-up period after the last vernakalant infusion or end of medical encounter (with at least 2 h of observation) seemed sufficient considering the short drug half-life and rapid distribution. However, since only short-term results were available and the final SR rate was not captured, no conclusion could be drawn concerning the effectiveness and outcomes beyond the acute phase. Finally, most patients (94.6%) were white, reflecting the patients' demographic characteristics in the seven European countries where the study was conducted. This could limit the external validity of the results in countries with large non-white immigrant populations (e.g., the UK) or other non-European populations. However, the impact of this limitation is probably limited because similar efficacy and safety results were obtained in patients with recent-onset AF treated with vernakalant in a phase 3 study in 123 patients from Korea, Taiwan, and India [34] and in previous trials conducted in other countries [20, 23]. This indicates a consistent effect of this drug in various populations.

In conclusion, this post hoc analysis of the SPECTRUM study showed that intravenous vernakalant for cardioversion of recent-onset AF was well tolerated with similar cardioversion rates in patients treated in the ED or non-ED setting and does not require admission to coronary care unit/intensive care unit. First-line treatment with vernakalant in the ED could shorten the duration of hospital stays for patients with recent-onset AF.

## Statement of Ethics

This study was mandated and approved by the European Committee for Medicinal Products for Human Use. The protocol was reviewed and approved by the Pharmacovigilance Risk Assessment Committee (PRAC; reference procedure EMEA/H/C/001215/MEA026.3). The protocol was approved by the appropriate local research Ethics Committees for all participating centres and the study was conducted in accordance with applicable national and local regulations, accepted standards for Good Clinical Practice, Guidelines for Good Pharmacoepidemiology Practices, and the Declaration of Helsinki. Informed written consent was obtained from all patients. The study is registered at http://www.clinicaltrials.gov (NCT01370629) and http://www.encepp.eu (EUPAS2078).

## Conflict of Interest Statement

Johan-Emil Bager and José L. Merino, via their respective institution, Alfonso Martín, José Carbajosa Dalmau, Alexander Simon, and Juha E.K. Hartikainen received payment from ADVANZ PHARMA for participation in the study. Alfonso Martín received advisory and speaker honoraria from Boehringer-Ingelheim, BMS-Pfizer, Cardiome, Medtronic, and Daiichi-Sankyo, as well as a grant from Bayer, outside of the submitted work. José Carbajosa Dalmau received a grant from FISABIO (Fundación para el Fomento de la Investigación Sanitaria y Biomédica de la Comunidad Valenciana) to participate in the study. Alexander Simon received a grant from the Austrian National Bank outside of the submitted work. José L. Merino received speaker fees from ADVANZ PHARMA Switzerland Sàrl, advisory board fees from Sanofi, grants from Daiichi-Sankyo and Bayer, and personal fees from Bayer, outside of the submitted work. Beate Ritz is an employee of ADVANZ PHARMA Switzerland Sàrl. Juha E.K. Hartikainen has received speaker honoraria from Amgen, Novartis, Sanofi Aventis, and AstraZeneca outside of the submitted work.

## Funding Sources

This work was supported by ADVANZ PHARMA Switzerland Sàrl, a company of the ADVANZ PHARMA group, previously known as Correvio International. The authors received no funding for their participation in the development of this publication.

## Author Contributions

Alfonso Martín contributed to the study development (since 2010), was country coordinator in Spain, contributed to hospital inclusions and investigator recruitment, country development, and patient enrolment control in Spain, was the principal investigator for the University Hospital Severo Ochoa, and contributed to patient recruitment and manuscript review. Johan-Emil Bager contributed to patient recruitment, was the principal investigator for the Sahlgrenska University Hospital, and contributed to the manuscript review. José L. Merino contributed to patient recruitment and was the principal investigator for La Paz University Hospital. José Carbajosa Dalmau, Alexander Simon, and Juha E.K. Hartikainen contributed to patient recruitment. Johan-Emil Bager, Alfonso Martín, José Carbajosa Dalmau, Alexander Simon, José L. Merino, Beate Ritz, and Juha E.K. Hartikainen contributed to the data interpretation and critically reviewed and approved the manuscript prior to submission.

## Data Availability Statement

The study results are available at http://www.clinicaltrials.gov (NCT01370629) and http://www.encepp.eu (EUPAS2078). The data that support the findings of this study are available from the corresponding author, upon reasonable request.

## Supplementary Material

Supplementary data

## Figures and Tables

**Table 1:** Summary of demographic characteristics of patients at the time of the vernakalant treatment episode in the ED and non-ED groups

	ED group^a^	Non-ED group^a^
	(*N* = 1,289)^b^	(*N* = 720)^b^
*Patient characteristics*		
Age, years	61.8±13.39	63.1±12.35
Male gender	760 (59.0)	462 (64.2)
Race^c^		
White	1,217 (94.6)	714 (99.2)
Black	3 (0.2)	2 (0.3)
Hispanic	6 (0.5)	0 (0.0)
Asian	1 (0.1)	1 (0.1)
Other	62 (4.8)	3 (0.4)
Body mass index,^d^ kg/m^2^	28.0±5.09	27.5±4.56
Systolic blood pressure,^e^ mm Hg	134.1±19.87	129.6±18.60
Heart rate,^f^ bpm	115.2±25.07	108.9±25.68

*Prior history*		
Hypertension	712 (55.7)	391 (54.5)
Diabetes	117 (9.1)	82 (11.4)
Angina	75 (5.9)	43 (6.0)
Cardiomyopathy	20 (1.6)	13 (1.8)
Hypertrophic obstructive	5 (25.0)	5 (38.5)
Restrictive	1 (5.0)	1 (7.7)
Other	14 (70.0)	7 (53.8)
History of HF	33 (2.6)	30 (4.2)
Stroke	60 (4.7)	31 (4.3)
Pacemaker/ICD	22 (1.7)	14 (2.0)

*Symptoms at baseline*		
Shortness of breath	188 (14.7)	164 (25.4)
Chest pain	175 (13.7)	96 (14.9)
Palpitations	1,188 (92.4)	561 (85.7)
Dizziness, light-headedness	207 (16.2)	113 (17.6)
Syncope/presyncope	40 (3.1)	21 (3.2)
Other	134 (10.4)	119 (16.5)

*Duration of current AFg*		
<3 h	105 (8.3)	65 (9.1)
3–24 h	893 (70.4)	375 (52.4)
24–48 h	178 (14.0)	169 (23.6)
>48 h	92 (7.3)	107 (14.9)
Mean (h)	19.26±40.62	30.27±50.92
Median (h)	9.12 [4.83, 19.29]	15.78 [7.73, 35.05]

*Use of antiarrhythmic drugsh*		
Beta-blockers	657 (51.0)	398 (55.3)
Calcium channel blockers	13 (1.0)	9 (1.3)
Class I antiarrhythmics^i^	64 (5.0)	21 (2.9)
Class III antiarrhythmics^j^	75 (5.8)	23 (3.2)
Digitalis glycosides	12 (0.9)	10 (1.4)

The values denote mean±standard deviation, *n* (%) of episodes in a given category, or median [IQR]. ED, emergency department; N, total number of episodes; bpm, beats per minute; ICD, implantable cardioverter-defibrillator; AF, atrial fibrillation.

a Missing values were not included in the percentage calculations.

b 1,289 episodes in 1,120 unique patients in the ED group and 720 episodes in 664 unique patients in the non-ED group.

c *N* = 1,287 in the ED group.

d *N* = 882 in the ED group and 615 in the non-ED group.

e *N* = 1,282 in the ED group and 715 in the non-ED group.

f *N* = 1,280 in the ED group and 717 in the non-ED group.

^g^ *N* = 1,268 in the ED group and 716 in the non-ED group. ^h^ Medications given within 24 h before hospital admission.

i Includes flecainide and propafenone.

j Includes amiodarone, dofetilide, and dronedarone.

**Table 2:** Pre-defined HOIs and other SAEs reported during the vernakalant infusion and the post-treatment observation period in patients who received vernakalant in the ED or an inpatient hospital setting

	ED group (*N* = 1,289)		Non-ED group (*N* = 720)	
	events^a^	drug related^a^	events^a^	drug related^a^
Pre-defined HOIs	**12 (0.9)**	**12 (0.9)**	**7 (1.0)**	**7 (1.0)**
Significant hypotension^b^	1 (0.1)	1 (0.1)	1 (0.1)	1 (0.1)
Any significant ventricular arrhythmia^c^	−	−	−	−
Significant bradycardia^b^	9 (0.7)	9 (0.7)	6 (0.8)	5 (0.7)
Significant AFL with 1:1 atrioventricular conduction^c^	2 (0.2)	2 (0.2)	−	−
Non-pre-defined SAEs	**5 (0.4)**	**4 (0.3)**	**4 (0.6)**	**1 (0.1)**
Hypotension	1 (0.1)	1 (0.1)	1 (0.1)	0 (0.0)
nsVT	1 (0.1)	1 (0.1)	1 (0.1)	1 (0.1)
SVT	1 (0.1)	1 (0.1)	−	−
Pericardial effusion	1 (0.1)	0 (0.0)	−	−
Vernakalant IV overdose	1 (0.1)	1 (0.1)	−	−
Angina pectoris	−	−	1 (0.1)	0 (0.0)
Visual disturbance	−	−	1 (0.1)	0 (0.0)

The values denote *n* (%) of episodes for which pre-defined HOIs/non pre-defined SAEs were reported. HOI, health outcome of interest; SAE, serious adverse event; ED, emergency department; N, total number of episodes; SVT, supraventricular tachycardia; nsVT, non-sustained ventricular tachycardia.

a Includes all events considered definitely, probably, or possibly related to intravenous vernakalant by the investigator.

b Both events of significant hypotension occurred simultaneously with significant bradycardia and therefore are reported in this table under both event types.

c One event was reported by the investigator as sustained VT with a differential diagnosis of AFL with 1:1 conduction. Following inspection of the patient's ECG, the event was re-categorized by the Safety Review Committee as significant AFL with 1:1 conduction.
