# Supplementary material for: Vernakalant for Cardioversion of Recent-Onset Atrial Fibrillation in the Emergency Department: The SPECTRUM Study
Source: Cardiology. 2022 Nov 10;147(5-6):566–77. doi: 10.1159/000526831 (PMC9808723; doi:10.1159/000526831)
Supplement: Supplementary file 1 — Supplementary data [file crd-0147-0566-s01.docx]

**Vernakalant for cardioversion of recent-onset atrial fibrillation in the emergency department: The SPECTRUM study**

*Johan-Emil Bager, Alfonso Martín, José Carbajosa Dalmau, Alexander Simon, José L. Merino, Beate Ritz, Juha E.K. Hartikainen*

**Online Supporting Information**

**Online Supplement S1.** Pre-infusion checklist


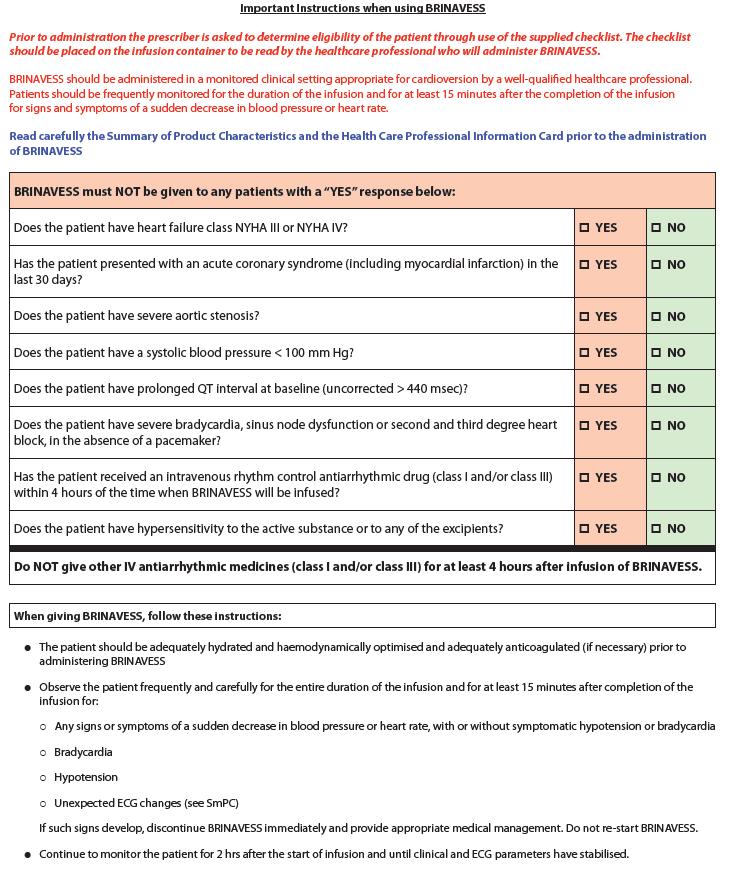


**Online Supplement S2.** Analyses performed on the effectiveness population

The per-protocol prespecified effectiveness population included all episodes, excluding those in patients who received electrical cardioversion or other pharmacological cardioversion within 90 minutes of the start of vernakalant infusion. In this population, successful cardioversion was determined according to the following definition: conversion to sinus rhythm for at least one minute within 90 minutes from the start of the first vernakalant infusion. A total of 1252 and 684 episodes were included in the effectiveness population in the emergency department (ED) and non-ED groups, respectively. In this population, successful cardioversion was reported in 70.2% (95% confidence interval [CI]: 67.5–72.7) and 71.0% (67.1–74.6) of patients in the ED and non-ED groups, respectively.

**Online Supplement S3.** Patients with single versus multiple inclusion(s) in the analysis in the ED and non-ED groups

| **Number of inclusions** | **ED group**  **(N=1120)** | **Non-ED group**  **N=664** |
| --- | --- | --- |
| **Single inclusion** | 995 (88.8%) | 619 (93.2%) |
| **Multiple inclusions** | 125 (11.2%) | 45 (6.8%) |
| **2** | 90 | 33 |
| **3** | 21 | 4 |
| **4** | 8 | 3 |
| **≥5** | 3 | 2 |

*Footnote*: ED, emergency department; N, number of patients. The values denote numbers (percentages) of patients.

**Online Supplement S4.** Atrial flutter (AFL) 1:1 events

One AFL 1:1 event occurred within two hours after the start of vernakalant infusion. The second AFL 1:1 event was reported in a patient who presented incomplete right bundle branch block at the electrocardiogram before vernakalant infusion. This patient did not convert after two vernakalant infusions. Three hours and six minutes after the start of vernakalant infusion, AFL with 1:1 conduction occurred with a maximum ventricular heart rate of 223 beats per minute and a duration of 300 seconds. The patient was hemodynamically unstable (with systolic blood pressure <90 mmHg) and underwent successful electrical cardioversion.

**Online Supplement S5.** Adherence to prescribed indications and dosage

Vernakalant was administered in 36/1289 (2.8%) and 65/720 (9.0%) episodes in patients with at least one contraindication in the emergency department (ED) and non-ED groups, respectively. In the ED group, the most frequently reported contraindications were the use of class I or class III/IV anti-arrhythmic drugs within four hours prior or subsequent to vernakalant infusion start (13 patients), baseline systolic blood pressure <100 mmHg (12 patients) and heart failure (seven patients). In one patient with contraindication (use of class III antiarrhythmics within four hours prior to vernakalant), a serious adverse event (SAE) of pericardial effusion considered unrelated to vernakalant was reported. No other health outcomes of interest (HOIs) or SAEs were reported among patients with available information on contraindications.

In patients with available information, vernakalant was administered with a dosage ranging between 95% and 105% of the weight-based recommendation in 1085/1184 (91.6%) and 650/714 (91.0%) patients for the first infusion, and in 427/465 (91.8%) and 273/293 (93.2%) patients for the second infusion in the ED and non-ED groups, respectively.

**Online Supplement S6.** Cardioversion effectiveness with vernakalant and length of ED stay in the SPECTRUM study and in published vernakalant cohort studies in the ED setting.


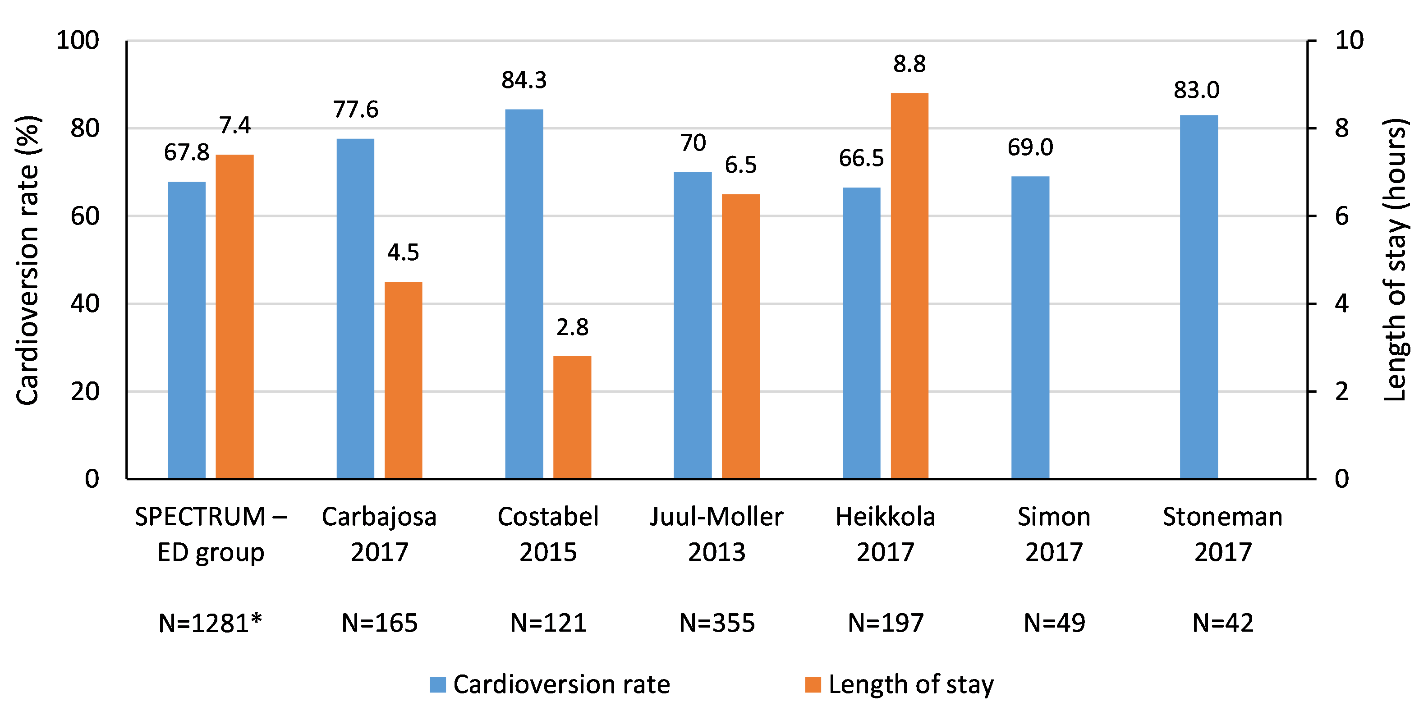


*Footnote*: ED, emergency department; N, number of patients with available results; SPECTRUM, Surveillance of Pharmacologic thErapy for Cardioversion in aTrial fibrillation Registry Using intravenous treatment; *Cardioversion data were missing for eight patients treated in the ED.
